# COVID-19 mortality with regard to healthcare services availability, health risks, and socio-spatial factors at department level in France: A spatial cross-sectional analysis

**DOI:** 10.1371/journal.pone.0256857

**Published:** 2021-09-17

**Authors:** Anastase Tchicaya, Nathalie Lorentz, Kristell Leduc, Gaetan de Lanchy

**Affiliations:** Luxembourg Institute of Socio-Economic Research, Living Conditions Department, Esch-sur-Alzette, Luxembourg; Sciensano, BELGIUM

## Abstract

**Background:**

The 2019 coronavirus (COVID-19) epidemic began in Wuhan, China in December 2019 and quickly spread to the rest of the world. This study aimed to analyse the associations between the COVID-19 mortality rate in hospitals, the availability of health services, and socio-spatial and health risk factors at department level.

**Methods and findings:**

This spatial cross-sectional study used cumulative mortality data due to the COVID-19 pandemic in hospitals until 30 November 2020 as a main outcome, across 96 departments of mainland France. Data concerning health services, health risk factors, and socio-spatial factors were used as independent variables. Independently, we performed negative binomial, spatial and geographically weighted regression models. Our results revealed substantial geographic disparities. The spatial exploratory analysis showed a global positive spatial autocorrelation in each wave indicating a spatial dependence of the COVID-19 deaths across departments. In first wave about 75% of COVID-19 deaths were concentrated in departments of five regions compared to a total of 13 regions. The COVID-19 mortality rate was associated with the physicians density, and not the number of resuscitation beds. Socio-spatial factors were only associated with the COVID-19 mortality rate in first wave compared to wave 2. For example, the COVID-19 mortality rate increased by 35.69% for departments densely populated. Health risk factors were associated with the COVID-19 mortality rate depending on each wave. This study had inherent limitations to the ecological analysis as ecological bias risks and lack of individual data.

**Conclusions:**

Our results suggest that the COVID-19 pandemic has spread more rapidly and takes more severe forms in environments where there is already a high level of vulnerability due to social and health factors. This study showed a different dissemination pattern of COVID-19 mortality between the two waves: a spatial non-stationarity followed by a spatial stationarity in the relationships between the COVID-19 mortality rate and its potential drivers.

## Introduction

The 2019 coronavirus (COVID-19) epidemic began in Wuhan, China in December 2019 and quickly spread worldwide [1]. COVID-19 disease is a respiratory infection caused by the severe acute respiratory syndrome coronavirus 2 (SARS-CoV-2) [2]. The World Health Organization (WHO) declared a state of global health emergency on 31 January 2020, and the global pandemic stage of the COVID-19 epidemic on 11 March 2020 [1]. In Europe, France was the second country, a week after Italy, to enter an epidemic phase. Beginning on 16 March 2020, several European countries took drastic measures to contain the spread of SARS-CoV-2 by containing their populations while also promoting the adoption of barrier gestures, such as social distancing, regular hand washing, and the use of hydro-alcoholic gel for hand disinfection. Due to the uncertainty associated with the spread of an unprecedented epidemic and limited capacities of the health system, the containment strategy became essential in France and several countries, to slow the growth of COVID-19 cases, avoid congestion in hospital structures, and mobilise the necessary resources.

The crisis precipitated by this pandemic exposed flaws in the health system, which experienced difficulties in coping with the rapid spread of the virus. During the first weeks of the COVID-19 epidemic in France the health system was quickly overwhelmed by the increased number of confirmed cases and related deaths, particularly in the Grand-Est and Île-de-France regions [3]. The chronic inadequacies of human and material resources the health system suffered for several decades were highlighted by the pandemic and resulted in organisational and logistical problems. In France, questions regarding masks, screening tests, reagents, mechanical respirators, and personnel protective equipment increased during the progressive unlock-down of the population that began on 11 May 2020. The health system had to employ the national medical reserve and medical students to supplement existing health personnel and strengthen response capacities against the spread of COVID-19. Nevertheless, the capacities in intensive care beds and resuscitation care have not been strengthened to anticipate a possible acceleration of the transmission of SARS-Cov-2 after the summer. The country has witnessed a worrying resumption of SARS-Cov-2 pandemic since September. The Government had adopted graduated measures to restrict the activities of bars and restaurants, as well as a curfew in the departments where intensive care and resuscitation services are approaching saturation. In November 2020, the government had decreed a curfew and lockdown throughout France when the pandemic had reached the second wave peak.

As of 30 November 2020, France declared 35 950 cumulative deaths in hospitals due to the COVID-19 pandemic, including 19 630 deaths from the first wave (up to 1 August) and 16 320 deaths from the second wave (2 August to 30 November 2020) [3]. The spatial distribution of mortality from the COVID-19 pandemic was uneven across departments, but the determinants of the unevenness were unknown. Were these spatial disparities in the COVID-19 mortality linked to the availability of health care services, socio-spatial factors or pre-existing health problems in the departments? As the causes of the pandemic spread and the fatal outcome of COVID-19 disease in infected individuals are multifactorial, several of those mentioned above are likely associated with the observed mortality rates.

Of course, over time, the scientific literature provides a global understanding of the various socioeconomic and spatial determinants of the incidence and mortality due to COVID-19 from an ecological analysis perspective [4–6]. In France, a study carried out by Semenzato et al. [7] found, not only age and gender (male) were the main factors of death from COVID-19, but almost all of the chronic diseases studied were positively associated with increased risks of hospitalisation for COVID-19 and hospital deaths.

The present study aims to analyse the associations between the number of COVID-19 deaths in mainland France, the availability of health services, the socio-spatial factors, and the prevalence of major health problems that are likely to explain the territorial disparities in the number of COVID-19 deaths observed at the department level. It will thus distinguish the strength of these associations in the first and second waves of the pandemic. This study also aims to put into perspective the strategic implications for combatting the COVID-19 pandemic in order to draw lessons for the future.

## Methods

### Design

This cross-sectional study utilised mortality data for deaths due to the COVID-19 pandemic recorded from the beginning of the pandemic until 30 November 2020 across the 96 departments of mainland France. During this period, we observed the two following epidemic waves in France: the first wave concerns the period from the start of the pandemic until 1 August 2020, and the second wave covers the period from 2 August to 30 November 2020. This last date limit is not necessarily the end of this wave from an epidemiological perspective.

### Data sources and data

Several data sources were used to carry out this study and all variables used are defined in Table 1. Data of healthcare services availability and socio-spatial characteristics at the department level was presented in Supporting information (S1 Table).

**Table 1:** Variables, definitions and data sources.

Variables	Definition	Data sources	Data year	Level considered
COVID-19 death rate in hospitals	Number of cumulated COVID-19 deaths in hospitals expressed per 100 000 people	Ministry of Health https://www.data.gouv.fr/fr/datasets/donnees-hospitalieres-relatives-a-lepidemie-de-covid-19 /	2020	Department
*Availability of healthcare services*				
Number of resuscitation beds	Number of resuscitation beds expressed per 100 000 people	Ministry of Health https://drees.solidarites-sante.gouv.fr	2018	Department
Physicians density	Number of physicians divided by the department area	Ministry of Health https://drees.solidarites-sante.gouv.fr	2018	Department
*Socio-spatial factors*				
%People aged 60+	Proportion of the population 60 years of age or older	Eurostat	2019	Department
%Males	Proportion of male sex in population	Eurostat	2019	Department
%Urban population	Proportion of people living in the greater urban areas (per cent).	FNORS/STATISS 2019, version of May 18, 2020	2016	Department
Rate of poverty	Rate of monetary poverty (per cent)	FNORS	2016	Department
Population density	Number of people by square kilometer	Eurostat	2019	Department
Population size	Size of population for each department (by thousand)	Eurostat	2019	Department
*Health risk factors*				
Stand_Diabetes	Standardised rates of people treated for diabetes (per 100,000 people).	https://www.ameli.fr/l-assurance-maladie/statistiques-et-publications/etudes-en-sante-publique/cartographie-des-pathologies-et-des-depenses/prevalence-departementale-par-pathologie/maladies-cardio-neurovasculaires-1ere-partie.php	2018	Department
Stand_Chronic heart failure	Standardised rates of people treated for chronic heart failure in 2018 per 100,000 people.	https://www.ameli.fr/l-assurance-maladie/statistiques-et-publications/etudes-en-sante-publique/cartographie-des-pathologies-et-des-depenses/prevalence-departementale-par-pathologie/maladies-cardio-neurovasculaires-1ere-partie.php	2018	Department
Stand_Chronic respiratory diseases (excluding cystic fibrosis)	Standardised rate of people treated for chronic respiratory diseases (excluding cystic fibrosis) (per 100,000 people).	https://www.ameli.fr/l-assurance-maladie/statistiques-et-publications/etudes-en-sante-publique/cartographie-des-pathologies-et-des-depenses/prevalence-departementale-par-pathologie/maladies-cardio-neurovasculaires-1ere-partie.php	2018	Department
*Geographic data*				
Latitude	Latitude of the department’s centroid			Department
Longitude	Longitude of the department’s centroid			Department

NB: The rate is standardised according to age, the reference population being the whole of France in the 2006 population census.

Cumulative mortality data due to the COVID-19 pandemic in hospitals were obtained from statistics from the French Ministry of Health. Data concerning the availability of health services (Number of resuscitation beds, number of intensive care beds, and medical density) at the departmental level were obtained primarily from the direction of Research, Studies, Evaluation and Studies (DREES) [8].

Data regarding socio-spatial factors, including demographic, socio-economic, and spatial characteristics were obtained from the European Statistical Office (EUROSTAT) and the National Federation of Regional Health Observatories (FNORS) [9].

Data on the prevalence of certain chronic diseases and health risk factors (diabetes, chronic respiratory diseases, and chronic heart failure) came from AMELI (www.ameli.fr).

#### COVID-19 mortality rate

The COVID-19 mortality rate in hospitals at the department level was the primary health outcome of this study and was defined as the ratio of the number of COVID-19 deaths to population size for 100 000 people.

#### Availability of health services

The availability of health services represents the ability of the health system to manage patients with severe forms of COVID-19. Here, it was defined in terms of the number of resuscitation beds, and medical personnel density at the department level. These data were defined per 100,000 people.

#### Socio-spatial factors

In this study, the socio-spatial factors included both demographic (proportion of people aged ≥60, proportion of males), socio-economic (proportion of unemployed adults, poverty rate, and spatial or environmental characteristics (population density, proportion of people living in the large urban areas). These socio-spatial factors were used separately in the analyses.

#### Health risk factors

Health risk factors included certain chronic diseases and risk factors such as diabetes, chronic heart failure, and chronic respiratory diseases (excluding cystic fibrosis). These health risk factors were selected due to their probable influence on the occurrence of severe or critical forms of COVID-19 among people with one or more health problems [9, 10]. These data were used to characterise the state of health and pathologies at risk of COVID-19 for people living in these departments. All health risk factors were calculated as age-standardised prevalence rates per 100 000 people.

#### Geographic data

The latitude and longitude of the centroid of the departments were used as geographical coordinates allowing us to measure the distance between two locations and investigate the spatial autocorrelation or spatial dependence.

### Statistical analysis

The characteristics of the dataset in terms of the number of COVID-19 deaths, health services availability, socio-spatial factors, and health status were described as means, standard deviations, minimum, and maximum values.

We performed a negative binomial regression analysis to assess the effects of health services availability, health risk factors, and socio-spatial factors on COVID-19 mortality rates using department-level COVID-19 deaths as the outcome. Negative binomial regression models allowed us to take account of overdispersion, which is often present in count data [11]. However, we also performed a negative binomial regression with overdispersion correction. Due to our small dataset size and regions with fewer than five departments, we did not perform a multilevel negative binomial analysis.

The results were assessed at a significant threshold of error equal to 5% or 95% confidence interval (95% CI). The parameter estimates from the negative binomial model were exponentiated to define the COVID-19 mortality rate ratios.

We also conducted a short exploratory analysis of departmental disparities in death rates due to COVID-19 by calculating the indices of Moran I and Geary c (and the Moran scatterplot). The Moran I index makes it possible to measure global spatial autocorrelation and to visualise the global spatial association scheme from the same name diagram [12, 13]. Thus, the spatial autocorrelation is positive if nearby places tend to resemble each other more than distant places; it is negative if the nearby places tend to be more different than the more distant places, and it is zero when no relation exists between the proximity of the places and their degree of resemblance [12, 13].

We also performed spatial models to evaluate spatial patterns in COVID-19 mortality such as Spatial Autoregressive (SAR) model, Spatial Error Model (SEM) and Geographically Weighted Negative Binomial Regression (GWNBR). GWNBR was used to highlight spatial heterogeneity by considering the spatial variability of the death rate due to COVID-19 compared to the potential determinants of the spread of the pandemic. We used the SAS macro developed by Silva and Rodrigues [14].

In addition, COVID-19 mortality maps were created to display the spatial disparities in the propagation of the COVID-19 pandemic across metropolitan France, using the PhilCarto and Inkspace free software. These maps also allowed us to visualise the variation of the COVID-19 mortality rate between the two different waves.

All analyses were performed using SAS (release 9.4) software (SAS Institute Inc., Cary, NC, USA), including global Moran’s I values and Moran scatterplot.

### Ethics statement

This study used aggregated data from government and public sources that are openly available. It did not need ethical approval in this case.

## Results

### Availability of healthcare service, socio-spatial and health risk characteristics

The average number of COVID-19 deaths in hospital per 100,000 people was 25.7 and 24.6 in the first and second waves, respectively (Table 2). The average number of resuscitation beds was 6.7 and 7.4, respectively. The average physicians density was 305 per department.

**Table 2:** Availability of healthcare services, socio-spatial and health risk characteristics of the departments, including COVID-19 deaths in waves 1 and 2, by mean (standard deviation), minimum and maximum.

	Mean (standard deviation)	Min	Max
COVID-19 deaths rate in hospitals (Wave 1, up to 1 August 2020)	25.7 (25.0)	1.3	142.7
COVID-19 deaths rate in hospitals (Wave 2, from 2 August to 30 November 2020)	24.6 (13.3)	4.3	67.7
Number of resuscitation beds (per 100,000)	6.7 (3.8)	2.0	21.9
Number of intensive care beds (per 100,000)	7.4 (4.6)	0.0	25.2
Physicians density (per 100,000)	305.0 (89.3)	167.0	858.0
% People aged 60+	29.6 (4.8)	16.7	39.3
% Males	48.5 (0.5)	47.0	49.6
% Unemployment	7.9 (1.6)	4.8	13.3
% Urban population (proportion of people living in the great urban areas)	71.2 (20.8)	0.1	100.0
Population density	565.8 (2425.1)	14.8	20459.7
Population size (by thousand)	676.0 (520.1)	76.3	2589.0
Stand_Chronic respiratory diseases (excluding cystic fibrosis) at the department-level (per 100,000)	1097.4 (137.9)	791.7	1454.6
Stand_Diabetes at the department-level (per 100,000)	1836.4 (292.2)	1257.4	2466.2
Stand_Chronic heart failure at the department–level t(per 100,000 people)	3958.0 (300.5)	3499.6	4514.6
Rate of poverty (per cent)	14.6 (3.1)	9.2	28.6

The social and spatial characteristics of the departments indicated that the population density was, on average, 566 people per square kilometre; 29.6% of the people were aged 60 and over, and 48.5% were men, while the average rate of unemployment reached 7.9%. On average, 71.2% of people lived in large urban areas. The mean age-standardised rates of chronic respiratory disease, diabetes, and chronic heart failure were 1097.4, 1836.4, and 3958.9, respectively.

### Georaphical distribution of the COVID-19 mortality rate in two waves

**Fig 1:**
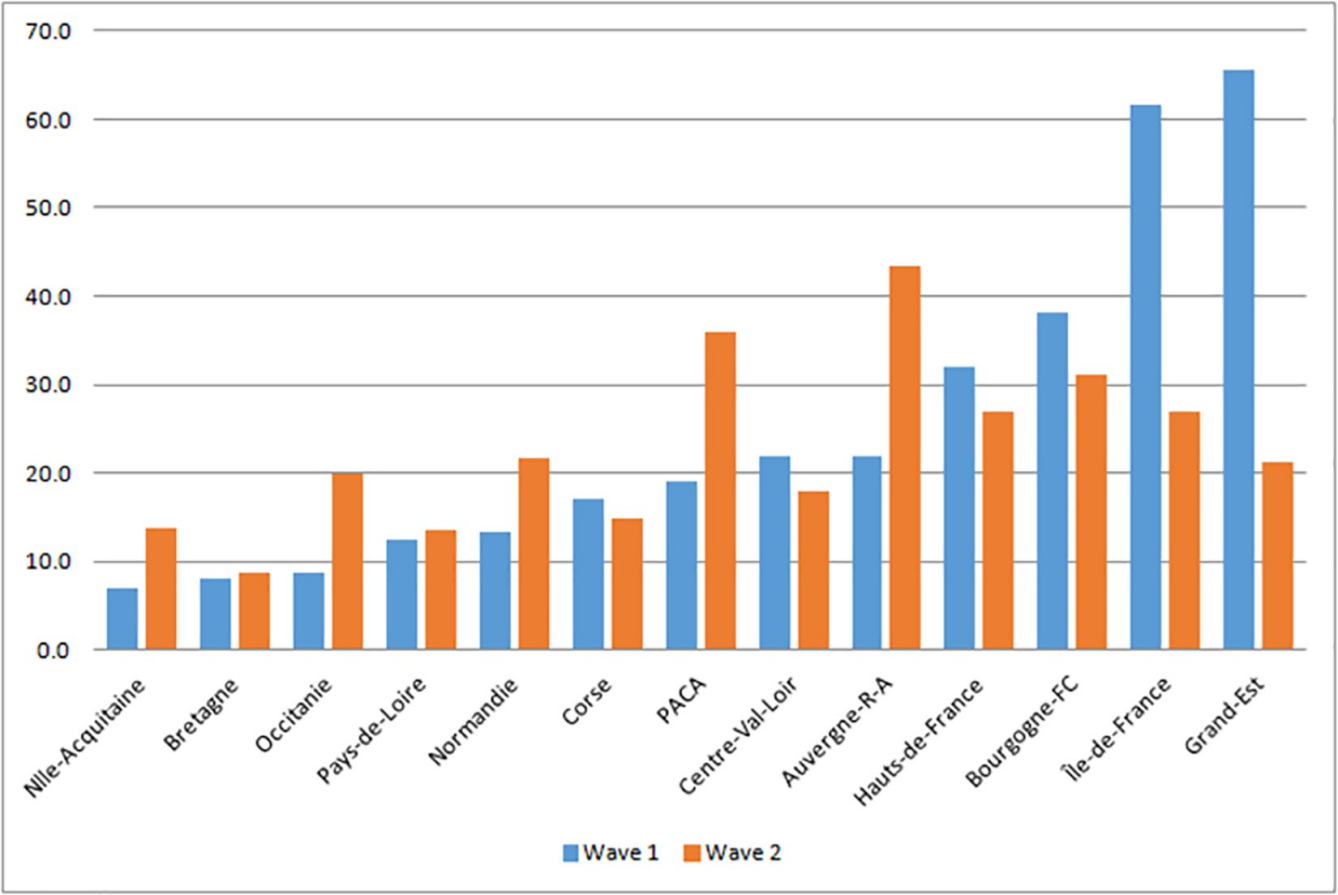
COVID-19 deaths rate in hospital (per 100,000 inhabitants), 30 November 2020.

The majority of deaths were concentrated in the departments located in the northern half and in the eastern of the country, respectively in wave 1 and wave 2 (Fig 1 and S1 Fig). The spatial disparities of the COVID-19 mortality rate in hospitals varied according to the availability of health services, health risk factors, and socio-spatial characteristics (S1 Fig).

The mortality rate was higher in the departments of four regions including Hauts-de-France, Bourgogne-Franche-Comté, Île-de-France, and Grand-Est during the first wave. During the second wave, the mortality rate was higher in four more regions compared to the first wave: Auvergne-Rhône-Alpes, PACA, Normandy, Occitanie and Nouvelle Aquitaine (Fig 1).

**Map 1:**
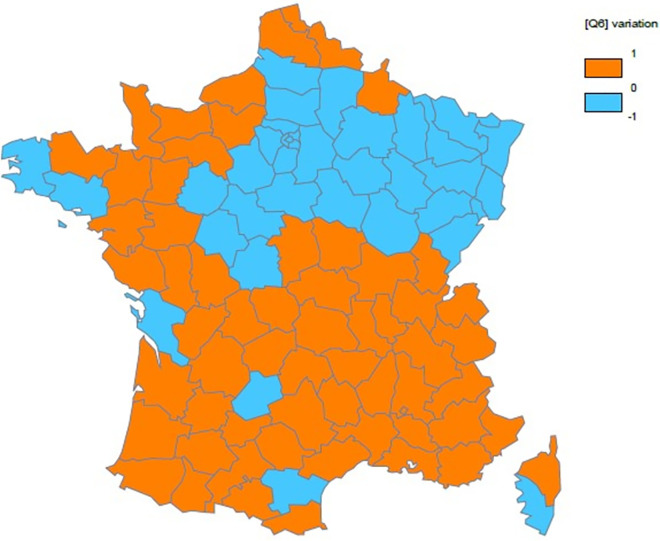
Spatial variation in the COVID-19 mortality rate gap between the first and second waves.

**Map 2:**
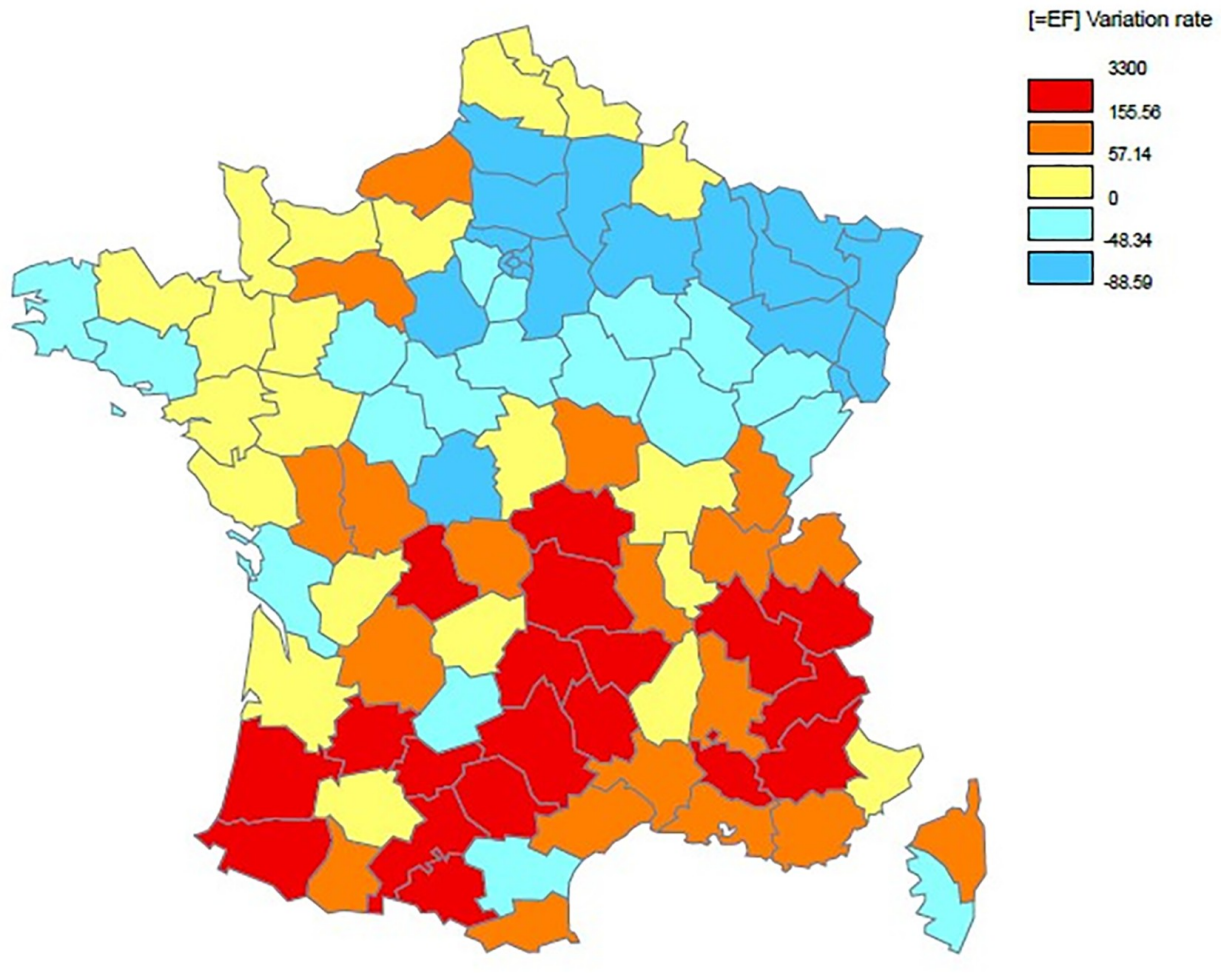
Spatial distribution of the change rate of the COVID-19 mortality rate between the first and second waves.

The Maps 1 and 2 below clearly illustrated variations in mortality rates between the two waves (S2 Table).

Map 1 shows that few departments observed a decrease (see blue color) of the COVID-19 mortality rate between the two waves.

Map 2 presents the spatial distribution of the change rate of the COVID-19 mortality rate between the two waves. In relative term, five departments have an increase of COVID-19 mortality rate from 481% to 3300%.

**Fig 2:**
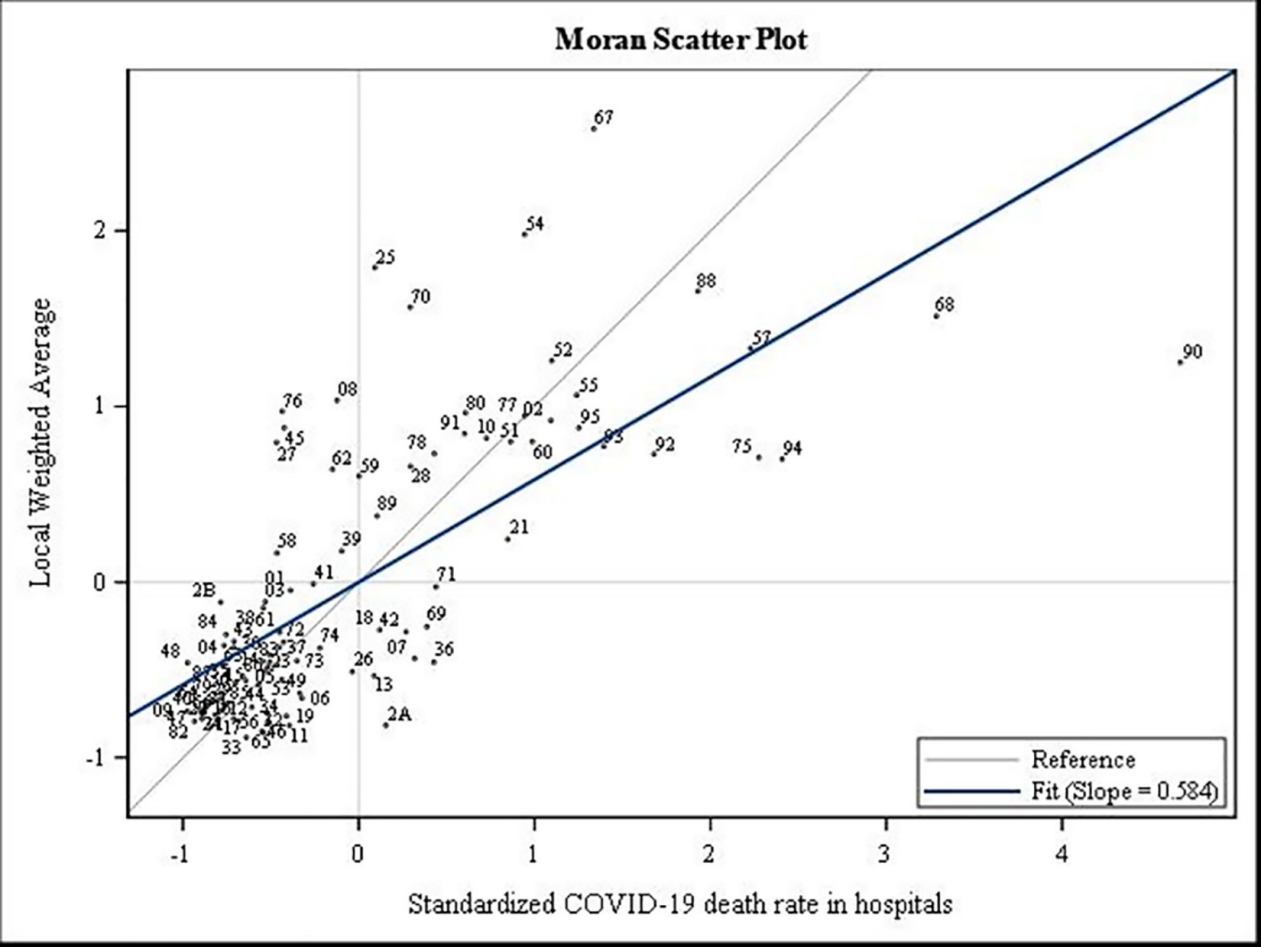
Spatial autocorrelation analysis for COVID-19 mortality rate in the first wave in 2020.

Furthermore, the exploratory spatial data analysis of the COVID-19 death rate in each wave revealed a spatial dependence measured through the Moran I and Geary c indices. The Moran I indicated a significant positive global spatial autocorrelation (Moran’s I = 0.626, p<0.0001; Moran’s I = 0.433, p<0.0001, respectively). The Moran I diagrams (Moran Scatterplot) below make it possible to visualise the overall spatial association diagram and identify some atypical departments such as the Territoires-de-Belfort (90), the Bas-Rhin (67), and the Haut-Rhin (68) in the first wave (Fig 2).

**Fig 3:**
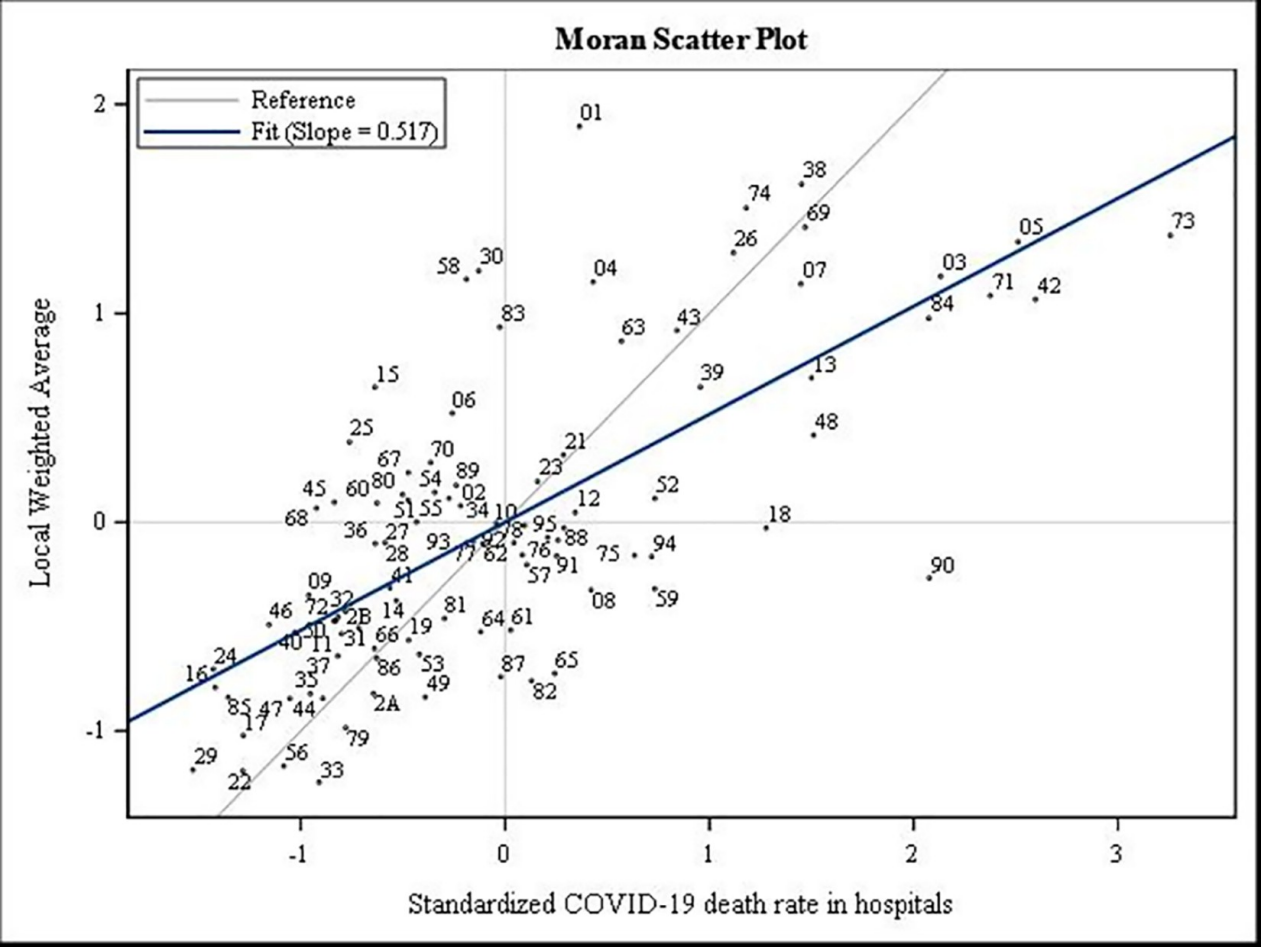
Spatial autocorrelation analysis for COVID-19 mortality rate in the second wave in 2020.

In the second wave, several departments from south-eastern have higher COVID-19 mortality rates (Fig 3).

By comparing the Moran’s I indices, the intensity of spatial dependence was strong in the first wave.

### Factors associated with the COVID-19 mortality rate for all 96 departments in France

The estimates of the negative binomial regression models assessing the potential associations of pandemic mortality with the availability of health services, population health risks, and socio-spatial factors at the department level are presented in Table 3.

**Table 3:** Estimates of effects of health services availability, socio-spatial factors, and health risk factors for COVID-19 pandemic mortality rate using the negative binomial regression model with over-dispersion correction (wave 1, wave 2 and overall period up to 30 November 2020).

	Wave 1	Wave 2	Overall period (full model)
	(full model)	(full model)	
Number of resuscitation beds (per 100,000 people)	1.0001	0.9989	0.9987
	(0.9960;1.0042)	(0.9962;1.0017)	(0.9961;1.0013)
Physicians density (per 100,000 people)	1.0017	1.0028**	1.0030**
	(0.9993;1.0042)	(1.0010;1.0047)	(1.0014;1.0046)
% People aged 60+	1.0811*	0.9988	1.0271
	(1.0115;1.1557)	(0.9505;1.0496)	(0.9829;1.0733)
% Males	1.6773**	1.2595	1.5210**
	(1.1625;2.4201)	(0.9368;1.6933)	(1.1807;1.9595)
% Urban population	1.0078	1.0035	1.0030
	(0.9945;1.0213)	(0.9942;1.0128)	(0.9946;1.0114)
Population density (log)	1.3569**	0.9474	1.1560
	(1.0870;1.6940)	(0.7955;1.1285)	(0.9920;1.3472)
Rate of poverty (per cent)	0.9207**	1.0213	0.9747
	(0.8650;0.9800)	(0.9776;1.0669)	(0.9359;1.0151)
Stand_Diabetes	1.0947***	1.0148	1.0525***
	(1.0686;1.1560)	(0.9962;1.0337)	(1.0355;1.0699)
Stand_Chronic heart failure	0.9850	1.1452*	1.0732
	(0.8422;1.1519)	(1.0207;1.2849)	(0.9661;1.1921)
Stand_Chronic respiratory diseases	0.9702**	0.9657***	0.9671***
	(0.9489;0.9920)	(0.9508;0.9809)	(0.9533;0.9811)

p<0.05 (*), p<0.001 (**), p<0.0001 (***)

#### Availability of healthcare services

The number of resuscitation care beds per 100,000 people was not associated with the COVID-19 mortality rate in hospitals in the waves 1 and 2, respectively (Table 3). Physician density was not associated with the COVID-19 mortality rate in hospitals in the wave 1, but significantly associated with it in the wave 2. However, the number of resuscitation care beds per 100,000 people and physician density were associated with the COVID-19 mortality rate in the wave 1 in Model 1 (S3 Table). In wave 2, only the physician density was associated with the COVID-19 mortality rate in models 2 to 4 (S4 Table).

#### Socio-spatial factors

The rate of poverty was associated with the COVID-19 mortality rate by a multiplier coefficient (mc) of 0.9293 or 7.07% decrease. The percentage of people living in large urban areas was associated with COVID-19 mortality rates in the first wave (mc = 1.0191 or 1.91% increase), but it was no longer associated with the COVID-19 mortality rate.

#### Health risks factors

Health risk factors such as diabetes, and respiratory diseases were significantly associated with the COVID-19 mortality rate in the first wave, with a coefficient of 1.0947 and 0.9702, respectively. In the second wave, cardiovascular and respiratory disease were significantly associated with the COVID-19 mortality rate (mc = 1.1452 and mc = 0.9657, respectively).

The other variables used for adjustment in the model, such as the proportion of people aged 60+ and share of males in the population, were significantly related to the number of COVID-19 deaths (mc = 1.0731 and mc = 1.7080, respectively) in the first wave, and those were no longer associated with the COVID-19 mortality rate in the second wave.

The results of the estimations from global spatial models of the SAR and SEM type have highlighted the significant positive influence of physician density, the share of people aged 60 and over, the share of men, the density of the population, and the prevalence of diabetes on the COVID-19 mortality rate overall period (Table 4). The results of the SAR and SEM spatial models confirmed the presence of autocorrelation of deaths due to COVID-19 (rho = 0.4655, p <0.0001; lambda = 0.5190, p <0.0001). In first wave, COVID-19 mortality rate was associated with the share of people aged 60 and over, the share of men, the density of the population, and the prevalence of diabetes. While in the second wave, only physician density was significantly associated with the COVID-19 mortality rate.

**Table 4:** Estimates of effects of health services availability, socio-spatial factors, and health risk factors for COVID-19 pandemic mortality rate using the SAR and SE models (including up to 30 November 2020).

	Wave 1		Wave 2		Overall period	
	SAR Model	SE Model	SAR Model	SE Model	SAR Model	SE Model
Number of resuscitation beds (per 100,000 people)	0.0024	0.0082	-0.0207	-0.0178	-0.0215	-0.0190
	(p = 0.9563)	(p = 0.8546)	(p = 0.4703)	(p = 0.5472)	(p = 0.7175)	(p = 0.7622)
Physicians density (per 100,000 people)	0.0425	0.0385	0.0418*	0.0319	0.0946*	-0.0899*
	(p = 0.1346)	(p = 0.1794)	(p = 0.0217)	(p = 0.0881)	(p = 0.0148)	(p = 0.0359)
% People aged 60+	2.7710**	3.3944***	-0.0477	0.0717	2.7527**	3.3174**
	(p = 0.0003)	(p<0.0001)	(p = 0.9248)	(p = 0.8984)	(p = 0.0085)	(p = 0.0050)
% Males	17.1197**	16.9540**	2.8682	2.1287	21.5450**	20.7752**
	(p = 0.0004)	(p = 0.0007)	(p = 0.3579)	(p = 0.5220)	(p = 0.0011)	(p = 0.0040)
% Urban population	0.2835	0.2252	0.0285	0.0164	0.3119	0.2363
	(p = 0.0591)	(p = 0.1575)	(p = 0.7717)	(p = 0.8763)	(p = 0.1228)	(p = 0.2792)
Population density (Log)	8.6069*	13.1061**	-0.1114	1.3302	8.9503*	13.9762**
	(p = 0.0016)	(p = 0.0006)	(p = 0.9481)	(p = 0.6127)	(p = 0.0132)	(p = 0.0035)
Rate of poverty (%)	-1.1174	-2.3260*	0.4413	0.7137	-0.8367	-1.9100
	(p = 0.0862)	(p = 0.0081)	(p = 0.2822)	(p = 0.2312)	(p = 0.3357)	(p = 0.0954)
Stand_Diabetes	1.1539**	1.5017*	-0.0123	-0.2115	1.3460**	1.7273**
	(p = 0.0004)	(p = 0.0016)	(p = 0.9448)	(p = 0.4947)	(p = 0.0018)	(p = 0.0078)
Stand_Chronic heart failure	-2.3791	-3.2347	1.9851	1.5671	-0.0904	-1.1192
	(p = 0.1784)	(p = 0.0924)	(p = 0.0915)	(p = 0.2229)	(p = 0.9698)	(p = 0.6818)
Stand_Chronic respiratory diseases	-0.2579	-0.4309	-0.3297	-0.1496	-0.7367*	-0.7993
	(p = 0.2913)	(p = 0.1631)	(p = 0.0605)	(p = 0.5107)	(p = 0.0344)	(p = 0.0775)
_lambda	-	0.6596***	-	0.7011***	-	0.5190***
		(p<0.0001)		(p<0.0001)		(p<0.0001)
_sigma2	184.6835	182.8419	79.0727	80.2623	337.7017	361.8009
	(p<0.0001)	(p<0.0001)	(p<0.0001)	(p<0.0001)	(p<0.0001)	(p<0.0001)
_rho	0.5511***	-	0.6172***	-	0.4655	-
	(p<0.0001)		(p<0.0001)		(p<0.0001)	
AIC	807.0157	810.1817	727.9340	733.1298	862.5242	870.5837
SBC	840.3522	843.5182	761.2706	766.4663	895.8607	903.9203
Log-vraisemblance	-390.5078	-392.0909	-350.9670	-353.5649	-418.2621	-422.2919

### Spatial distribution of the relationship between COVID-19 mortality rate and its driver factors using Geographically Weighted Negative Binomial Regression

The use of the GWNBR applied to the full model showed a spatial heterogeneity in the relationship between the COVID-19 mortality rate and the factors considered except for the physician density during the first wave (Table 5, wave 1). The interquartile range (IQR), defined as the range between the first quartile and third quartile of estimated coefficients of GWNBR model, was twice as large as the standard error in the negative binomial regression model. The Pseudo R^2^ was equal to 0.955, which corresponded to 95.5%, explaining the relationships observed between the COVID-19 mortality rate and the independent variables.

**Table 5:** Summary of parameter estimates of GWNBR models and assessing for spatial heterogeneity (wave 1).

Parameters	Minimum	1^st^ Quartile	Median	3^rd^ Quartile	Maximum	Interquartile (IQR)	Standard Error	Status
Intercept	-106.8916	-24.55988	-9.84323	1.42885	46.47659	25.98874	10.0613	Non-stationary
Number of resuscitation beds (per 100,000)	-0.00747	0.00019	0.00359	0.00517	0.01446	0.00498	0.0021	Non-stationary
Physicians density	-0.00783	-0.00072	0.00035	0.00160	0.00680	0.00232	0.0012	Stationary
% People aged 60+	-0.06835	-0.00826	0.07492	0.16215	0.30724	0.17041	0.0340	Non-stationary
% Males	-0.69494	-0.00570	0.15364	0.47115	1.88999	0.47685	0.1871	Non-stationary
% Urban population	-0.03044	-0.01328	0.00472	0.01770	0.07214	0.03098	0.0068	Non-stationary
Population density (Log)	-1.59995	-0.00317	0.20505	0.35655	1.87409	0.35972	0.1132	Non-stationary
Rate of poverty (%)	-0.37924	-0.12368	-0.04306	0.02506	0.21311	0.14874	0.0318	Non-stationary
Stand_Diabetes	-0.13867	0.03601	0.06568	0.09388	0.24011	0.05787	0.0123	Non-stationary
Stand_Chronic heart failure	-0.44709	-0.16138	-0.01496	0.13810	0.29737	0.29947	0.0799	Non-stationary
Stand_Chronic respiratory diseases	-0.11869	-0.06325	-0.04230	-0.02685	0.01147	0.03640	0.0113	Non-stationary

Pseudo R^2^ (pctdev) = 0.9550, Adjusted R^2^ = 0.8786, BIC = 885.8180, AIC = 729.9016, AICC = 949.6566. p = 0.0090, t-critical = 2.76

The relationship between the COVID-19 mortality rate and its drivers was stationary during wave 2 (Table 6, wave 2) and over the entire period considered (S5 Table, period up to 30 November 2020). The Pseudo-R^2^ was equal to 0.3535, which indicated that the model only explained 35.35% of the relationships of the COVID-19 mortality rate and its drivers.

**Table 6:** Summary of parameter estimates of GWNBR models and assessing for spatial heterogeneity (wave 2).

Parameters	Minimum	1^st^ Quartile	Median	3^rd^ Quartile	Maximum	Interquartile (IQR)	Standard Error	Status
Intercept	-9.66602	-9.23565	-9.05679	-8.86684	-8.48527	0.36881	8.1914	Stationary
Number of resuscitation beds (per 100,000 people)	-0.00115	-0.00104	-0.00098	-0.00094	-0.00086	0.00010	0.0014	Stationary
Physicians density	0.00261	0.00270	0.00276	0.00282	0.00294	0.00012	0.0009	Stationary
% People aged 60+	-0.00403	-0.00267	-0.00172	-0.00098	0.00023	0.00169	0.0253	Stationary
% Males	0.21429	0.22335	0.22734	0.23168	0.24104	0.00833	0.1510	Stationary
% Urban population	0.00328	0.00343	0.00353	0.00361	0.00375	0.00019	0.0047	Stationary
Population density (Log)	-0.05982	-0.05768	-0.05651	-0.05536	-0.05203	0.00232	0.0892	Stationary
Rate of poverty (%)	0.01838	0.02004	0.02155	0.02258	0.02364	0.00254	0.0223	Stationary
Stand_Diabetes	0.01365	0.01417	0.01446	0.01473	0.01534	0.00056	0.0094	Stationary
Stand_Chronic heart failure	0.12645	0.13390	0.13708	0.14144	0.14906	0.00754	0.0587	Stationary
Stand_Chronic respiratory diseases	-0.03591	-0.03568	-0.03543	-0.03517	-0.03486	0.00052	0.0079	Stationary

Pseudo R^2^ (pctdev) = 0.3535, Adjusted R^2^ = 0.2632, BIC = 760.3106, AIC = 727.9011, AICC = 732.0868, p = 0.04352, t-critical = 2.05

## Discussion

The COVID-19 pandemic unevenly affected the departments of France. Specifically, the departments of the Grand-Est, Île-de-France, Auvergne-Rhône-Alpes, and Hauts-de-France regions were affected most, representing 75% of deaths due to the COVID-19 pandemic during the first wave. During the second wave, departments in previously low-impact regions had recorded the highest death rates. The exploratory analysis of inequalities between the departments revealed the existence of a significant and positive global spatial autocorrelation. This result assumes that the departments were associated with a relatively high mortality rate (respectively relatively low) more often than if this location was purely random. Thus, spatial autocorrelation measures the intensity of the relationship between the proximity of places and their degree of resemblance [12].

### Association of the COVID-19 mortality rate with availability healthcare services

Containment measures and social distancing measures helped curb the spread of the COVID-19 pandemic and smooth the curve of admissions and deaths in hospital resuscitation and intensive care units. As the health system was quickly overwhelmed due to a chronic insufficiency of material (resuscitation beds, protective equipment for personnel, breathing apparatus) and human resources, the French government authorised hospitals and private clinics to increase their capacities in intensive care units across the country. Thus, the total capacity of intensive care beds increased from 5,000 to 8,000 beds on 24 March 2020 [15]. This deployment of resources in response to the rapid spread of the pandemic could explain the absence of statistically significant links between the number of COVID-19 deaths and the number of resuscitation beds considering the full model over both the two waves and the entire period. Several studies have not found significant associations between the availability of health care resources and the death rate from COVID-19 [16–18]. Other studies have found that the number of intensive care beds and the number of general practitioners per 10,000 people were associated with aggregated hospital fatality rate due to COVID-19 [19]. Nevertheless, there was a statistically significant positive association between the number of resuscitation beds and the COVID-19 mortality rate in model 1 of the first wave without adjustment with other covariates (S3 Table). However, this increase in the capacity of intensive care and resuscitation beds has come at the expense of patients suffering from other pathologies for which care and surgical procedures have been postponed. Physician density was significantly associated with the COVID-19 mortality rate in the second wave after adjusting for other covariates. The GWNBR model revealed a spatially non-stationary relationship between the COVID-19 mortality rate and the resuscitation beds rate during the first wave and spatially stationary during the second wave. In contrast, the relationship between COVID-19 death rate and physician density was stationary during the two waves.

Furthermore, the absence of statistically significant links between the availability of health resources and the death rate due to COVID-19 could be explained in part by the effect of the adoption of various measures aimed at controlling the disease and its consequences on the congestion of hospital services (lockdown, curfew). To this, we could add the transfer of COVID-19 patients to departments less impacted by the pandemic or the mobility of health personnel to other departments to support their colleagues in departments under pressure. The type of modelling used could also influence these results, particularly the so-called global models (non-spatial or spatial).

### Social and spatial-related factors that influence the mortality rates throughout France

The demographic, socioeconomic, and environmental factors, including the health risk behaviours they induce, contribute to social and geographic disparities in health status. The first studies published on the demographic characteristics of people with severe forms of COVID-19 revealed that people aged ≥60 and men represented a greater proportion of the victims than young adults and women [20, 21]. Our results were consistent with those from the analysis of clinical data from patients affected by COVID-19 during the first wave. The COVID-19 mortality rate is multiplied by 1.0811 and 1.6773, respectively, in the departments that have a high proportion of people aged ≥60 and males in their population. In other words, one percentage point increase in the proportion of people aged ≥60 was associated with an increase in the COVID-19 mortality rate of 8.11%. Likewise, one percentage point increase in the proportion of males in the population was associated with a 67.73% increase in the COVID-19 mortality rate. The difference in the risk of death due to COVID-19 between men and women could partly be explained by the higher prevalence of health problems among men than women, including diseases such as cardiovascular disease, diabetes, and arterial hypertension, which are risk factors for severe COVID-19 [22]. However, with the pandemic spreading more widely across the country, neither the share of people aged 60+nor the share of males was stillstatistically associated with the death rate from COVID-19. Over the entire period, only the share of men in each department was associated with the death rate due to COVID-19 (mc = 1.5210).

Over the entire period studied, our results showed that the percentage of the urban population, the population density (expressed in logarithm) and the poverty rate were not significant. However, these results hided time and space-related differences across the waves of the coronavirus spreading in the country. Thus the population density (mc = 1.3569) and the poverty rate (mc = 0.9207) were significant during the first wave. Amdaoud et al. [23] also found a significant link between population density and the death rate due to COVID-19 in hospitals in France based on data collected on 12 April 2020.

The population density was the strongest predictor of the variations of the COVID-19 mortality rate among departments [4]. However, the absence of significant relationships between population density and COVID-19 mortality rate in the second wave or overall studied period could be explained by the spread of the pandemic to more departments less densely populated as rural areas.

The GWNBR model coefficients of socio-spatial factors were spatially varied in the first wave because their IQRs were at least twice as large as the standard errors of the corresponding negative binomial model coefficient [24]. These results indicated that the associations between COVID-19 mortality rate and socio-spatial factors were non-stationary across the departments in the first wave. Therefore, the relationships between COVID-19 mortality rate and socio-spatial factors were stationary in the second wave.

### Pre-existing health conditions associated with a higher risk of mortality rate due to the COVID-19

Increased understanding of COVID-19 suggests that severe and critical forms of the disease are manifested more frequently among people with at least one comorbidity, regardless of age [10, 21]. This is an essential finding from this study that may aid in the preparation of strategies to fight the pandemic, from the prevention of infection to the care of COVID-19 positive patients.

Our analyses provided over-dispersion-corrected results that were consistent with those observed with individual and recent clinical data. Indeed, the COVID-19 mortality rate was significantly associated with the standardised rate of prevalence for chronic respiratory disease and the prevalence for diabetes during the considered period up to 30 November 2020. However, in the second wave, the prevalence rate for chronic heart disease was significant and not the prevalence rate for diabetes. A review of severe COVID-19 hospitalised cases or deaths revealed that the presence of cardiovascular disease was often a common comorbidity [25]. The relationship between the COVID-19 mortality rate and the prevalence rate for chronic respiratory disease was common to the two waves in our study. This result was not consistent with clinical studies on asthma and other respiratory pathologies [26, 27]. In a recent review of the literature, Morais-Almeida et al. emphasised the following: ‘There is no strong evidence supporting [the idea] that patients with asthma have a higher risk of becoming seriously ill from COVID-19’ (p.680) [26].

Diabetes has often been identified among patients with severe or critical forms of COVID-19, and even death, in Asia, Europe, and the United States [28]. In Italy, 35.5% of the deaths from COVID-19 out of a sample of 355 were people with diabetes [21]. In our study, the prevalence of diabetes was significantly associated with the COVID-19 mortality rate during the first wave and did not in the second wave. However, this association was statistically significant considering all the period of this study.

Zaldo-Aubanell et al. [29] showed an increased risk for COVID-19 mortality in areas with more per cent people with cardiovascular disease. Our results highlight the consequences of territorial inequalities in health care in the face of a COVID-19 pandemic, as well as the differences in dynamics regarding the severity of the COVID-19 disease in space and time. These findings have significant practical implications from a planning and resource allocation perspective.

### Practice implications

Public decision-makers need to meet numerous organisational, material, and logistical challenges to prepare health systems to better cope with future pandemics. To do so, an adjustment of the managerial approach is required in France and many other European countries, given the difficulties that have arisen in the fight against the COVID-19 pandemic. The mobilisation of material and human resources takes time, especially in crisis and scarcity contexts. One of the lessons learned from the COVID-19 experience is the need to enact prompt population-based measures, including social distancing, quarantine, and patient isolation actions to flatten the curve of the epidemic [30], testing and the use of masks, and assigning priority to the elderly and those suffering from chronic pathologies. In order to allow the health system to be flexible, a transparent ethical framework must be implemented that takes into account both the principles of maximising benefits and the equitable distribution of resources to ensure its acceptance by society [30–32]. The WHO admitted that governments would be unable to provide adequate care for their entire populations during a pandemic when health resources are limited [31]. Currently, legislation related to the liberties and rights of people could act as an obstacle to the effective application of people-tracking measures to fight the pandemic. An effective monitoring system capable of obtaining objective and comparable data that are crucial for determining the relative effectiveness of the various national strategies used is also necessary [33]. As OECD underscores, the health system needs to strengthen capacity at hospital and intensive care levels and the skills of health professionals in treating epidemic diseases [34].

The results of this study suggest that the COVID-19 pandemic quickly spread and developed severe and critical forms in environments where the population had a relatively unfavourable epidemiological picture. This finding was particularly strong during the first wave of the spread of the pandemic across mainland France. The spatial distribution of the relationships between the COVID-19 mortality rate and the various factors considered was dominated by non-stationarity, while during the second wave, they were stationary.

Improved knowledge of the social and epidemiological characteristics of people who developed severe forms of COVID-19 should enable public decision-makers and those in charge of the health system to develop effective strategic plans to fight epidemics and pandemics that are adapted to each context by allocating material, human, and financial resources equitably and optimally. In our study, the use of data aggregated at the department levels confirmed the existence of statistically significant relationships between COVID-19 mortality rates, availability of health services, pre-existing health risk factors in the population, and socio-spatial factors.

### Limitations

This study presents some limitations associated with its cross-sectional and ecological design. Therefore, the results should be interpreted with caution as these are the association relationships and not the causal relationships between the COVID-19 mortality rate and its potential determinants. Some limitations are partly related to the absence of certain factors that may also help explain the mortality rate due to the COVID-19 pandemic at the department level. We did not employ individual data or data on behavioural risk factors at the aggregate level, such as the percentage of people who are obese, the percentage of smokers people, the proportion of housing with poor quality (or no safety) at the department level. Previous studies report a significant association between the prevalence of obesity and hospitalised patients with severe COVID-19 in France [35, 36]. However, we were constrained by the sample size with 96 departments in mainland France. Additionally, we were unable to take into account the adjustments made in terms of the allocation of additional human and material resources as the COVID-19 pandemic progressed, particularly in the departments most affected.

This study did not seek to measure the impact of measures adopted by the government at national or local levels to curb the saturation of resuscitation services, the spread of the pandemic and deaths from COVID-19. Because these measures were not the objective of this study and were often national in scope throughout the country (lockdown during the first and second waves, the establishment of curfews) or sometimes they were first local before being generalised shortly after their implementation. This variation of decisions could make it difficult to measure the specific impact of the various measures.

Finally, this study found that the results with a non-spatial model as negative binomial regression were similar with those with a spatial model as SAR model. Using a GWNBR revealed the nature of the spatiality of relationships between COVID-19 mortality rate and its potential determinants. Compared to global spatial models (SAR and SEM), GWNBR was better in capturing the spatial heterogeneity with lower AIC for each wave.

Future research is expected to incorporate environmental factors such as temperature and humidity, which have negative associations with the COVID-19 cases [37], as well as air pollution, which may promote the spread of COVID-19 [38–40]. The resurgence of contamination’ cases and subsequent hospitalizations since the beginning of September in France and Europe, after a lull in June to August, encourages further investigations into the role of these environmental factors in the spread of SARS-Cov-2.

## Conclusion

This study revealed statistically significant links between COVID-19 mortality rates, the availability of health services, and health risk factors across the 96 departments of France. Departments with a high percentage of people aged ≥60, men, density population, people with diabetes had higher COVID-19 mortality rates in the first wave. While in the second wave, departments with more people with chronic CVD disease registered higher death rates. Globally, this study showed a different dissemination pattern of COVID-19 and its consequences in terms of mortality between the two waves. The first wave was characterised by a spatial heterogeity (or non-stationarity) in the relationships between the COVID-19 mortality rate and its potential determinants. In the second wave, these relationships were stable or stationary. Our results suggest that the COVID-19 pandemic has spread more quickly and took severe forms in departments where there were already a high prevalence of chronic health conditions, densely populated areas. Given these results, it is necessary to develop strategies to fight against epidemics, taking into account the degree of exposure of populations to specific chronic pathologies, some of which are linked to individuals’ behaviour and living conditions.

## Supporting information

S1 Table Availability of healthcare services and socio-spatial characteristics at the department level. (PDF)

S2 Table Difference of the COVID-19 mortality rate in hospital between the first and second waves. (PDF)

S3 Table Estimates effects of health services availability, socio-spatial factors, and health risk factors for COVID-19 mortality rate using the negative binomial regression model with over-dispersion correction. (PDF)

S4 Table Estimates effects of health services availability, socio-spatial factors, and health risk factors for COVID-19 mortality rate using the negative binomial regression model with over-dispersion correction. (PDF)

S5 Table Summary of parameter estimates of GWNBR models and assessing for spatial heterogeneity (overall period up to 30 November 2020: wave 1 and wave 2 included). (PDF)

S1 Fig Spatial disparity of the COVID-19 mortality rate in hospital according to healthcare services availability and health risk at the department level between the first wave and second wave in 2020. (PDF)

S2 Fig Spatial disparity of the COVID-19 mortality rate in hospital according to socio-spatial factors at the department level between the first wave and second wave in 2020. (PDF)

S3 Fig Spatial autocorrelation analysis for COVID-19 mortality rate in the first two waves. (TIF)

## Abbreviations

AIC: Akaike Information Criterion
AICC: Akaike Information Criterion Correction
BIC: Bayesian Information Criterion
COVID-19: 2019 Coronavirus
DREES: Direction of Research, Studies, Evaluation and Studies
EUROSTAT: European Statistical Office
FNORS: National Federation of Regional Health Observatories
GWNBR: Geographically Weighted Negative Binomial Regression
IQR: Interquartile Range
SARS-CoV-2: Severe Acute Respiratory Syndrome Coronavirus 2
SAR: Spatial Autoregressive
SEM: Spatial Error Model
WHO: World Health Organization
