# Supplementary material for: COVID-19 mortality with regard to healthcare services availability, health risks, and socio-spatial factors at department level in France: A spatial cross-sectional analysis
Source: PLoS One. 2021 Sep 17;16(9):e0256857. doi: 10.1371/journal.pone.0256857 (PMC8448369; doi:10.1371/journal.pone.0256857)
Supplement: S1 Table — (PDF) [file pone.0256857.s001.pdf]

S1 Table: Availability of healthcare services and socio-spatial characteristics at the department level

Department		Region name	Number of resuscitation beds (per 100,000 people)	Number of intensive care beds (per 100,000 people)	Medical density (per 100,000 people)	Population density (per 100,000 people)	Population size (by thousand)	% People aged 60 or more	% men	% Unemployment	% Urban population	Rate of poverty (per cent)
number	name											
1	Ain	Auvergne-Rhône-Alpes	22	10	186	114·0	657·0	24·2	49·2	5·9	86·7	10·7
2	Aisne	Hauts-de-France	34	55	230	71·4	526·1	27·9	48·9	11·4	69·8	18·9
3	Allier	Auvergne-Rhône-Alpes	24	28	285	45·1	331·3	35·6	47·8	8·8	67·2	15·5
4	Alpes-de-Haute-Provence	Provence-Alpes-Côte d'azur	6	0	304	23·9	165·2	34·3	48·8	9·8	41·6	16·4
5	Hautes-Alpes	Provence-Alpes-Côte d'azur	8	0	441	25·5	141·8	32·4	48·8	7·6	45·7	14·3
6	Alpes-Maritimes	Provence-Alpes-Côte d'azur	94	91	461	251·1	1079·4	31·3	47·2	8·5	98·6	15·7
7	Ardèche	Auvergne-Rhône-Alpes	8	0	242	59·1	326·9	32·7	48·8	9	63	14·4
8	Ardennes	Grand-Est	12	21	239	50·8	265·5	29·7	48·6	9·9	61·6	19·4
9	Ariège	Occitanie	8	8	284	31·2	152·4	34·4	49·0	10·3	35·1	18·5
10	Aube	Grand-Est	12	18	234	51·6	309·9	28·3	48·4	10·2	65·8	16·2
11	Aude	Occitanie	16	22	297	60·7	372·7	34·1	48·0	10·9	60·9	21·1
12	Aveyron	Occitanie	11	16	267	31·9	278·4	35·3	49·3	6·2	34·4	13·7

13	Bouches-du-Rhône	Provence-Alpes-Côte d'azur	309	238	444	399·9	2034·5	26·9	47·8	9·6	98·8	18·5
14	Calvados	Normandie	99	64	367	124·6	691·5	29·1	47·9	7·5	88·7	12·6
15	Cantal	Auvergne-Rhône-Alpes	8	15	290	24·9	142·8	37·0	48·9	4·8	45	13·4
16	Charente	Nouvelle-Aquitaine	12	18	258	58·5	348·2	33·4	48·3	7·9	70·7	14·9
17	Charente-Maritime	Nouvelle-Aquitaine	30	36	311	94·3	647·1	35·9	47·8	8·3	68·8	13·4
18	Cher	Centre-Val-de-Loire	12	16	212	41·0	296·4	33·9	48·4	8·5	67·5	14·9
19	Corrèze	Nouvelle-Aquitaine	15	21	279	41·0	240·3	35·3	48·5	6·6	60·7	13·2
21	Côte-d'Or	Bourgogne-Franche Comté	63	95	323	60·8	532·9	27·9	48·1	6·5	84·7	11·6
22	Côtes-d'Armor	Bretagne	14	16	291	86·7	596·2	34·1	48·2	7·1	61	12·1
23	Creuse	Nouvelle-Aquitaine	8	8	389	20·9	116·3	39·3	48·6	7·7	27·5	18·4
24	Dordogne	Nouvelle-Aquitaine	19	18	266	45·1	408·4	37·8	48·3	8·4	47	16·5
25	Doubs	Bourgogne-Franche Comté	43	131	234	103·1	539·4	26·2	48·9	7·5	77·3	12·2
26	Drôme	Auvergne-Rhône-Alpes	24	34	238	79·7	520·6	28·9	48·4	9	73·4	15·2
27	Eure	Normandie	12	22	359	99·5	600·7	26·5	48·7	8·1	80·6	13
28	Eure-et-Loir	Centre-Val-de-Loire	20	22	298	73·0	429·4	27·7	48·8	7·8	78·4	12·2
29	Finistère	Bretagne	69	71	167	134·6	906·6	30·7	48·6	7	58·2	10·8

2A	Corse-du-Sud	Corse	10	15	214	40·5	162·4	31·1	48·3	7·7	69·2	17·2
2B	Haute-Corse	Corse	8	6	342	39·1	182·3	30·4	48·5	8·7	55·4	21·7
30	Gard	Occitanie	49	54	311	127·9	748·5	30·5	48·1	11·4	84·3	19·8
31	Haute-Garonne	Occitanie	165	205	424	222·1	1400·9	22·0	48·6	7·8	96·2	12·8
32	Gers	Occitanie	8	4	245	30·4	190·0	36·1	48·7	6·1	38·9	15
33	Gironde	Nouvelle-Aquitaine	195	131	422	163·7	1633·4	25·3	48·1	7·7	88·5	12·7
34	Hérault	Occitanie	128	171	440	192·8	1176·1	28·0	47·7	11·6	83·4	19·2
35	Ille-et-Vilaine	Bretagne	66	112	344	159·7	1082·1	23·9	48·8	6·2	91·4	10·6
36	Indre	Centre-Val-de-Loire	10	9	217	32·0	217·1	36·6	48·1	7·9	41·9	14·8
37	Indre-et-Loire	Centre-Val-de-Loire	86	58	382	98·8	605·4	28·6	48·0	7·5	84·4	12·8
38	Isère	Auvergne-Rhône-Alpes	82	89	344	170·2	1265·0	24·8	49·0	6·6	93·3	11·5
39	Jura	Bourgogne-Franche Comté	8	9	242	51·6	257·8	31·3	48·6	5·9	55·6	11·7
40	Landes	Nouvelle-Aquitaine	14	16	276	44·6	412·0	33·0	48·3	8	48·6	11·9
41	Loir-et-Cher	Centre-Val-de-Loire	12	8	242	51·7	327·8	32·3	48·5	6·7	61·9	12·8
42	Loire	Auvergne-Rhône-Alpes	78	60	339	160·0	764·7	28·8	48·3	8·1	88·5	14·9
43	Haute-Loire	Auvergne-Rhône-Alpes	8	10	226	45·6	226·9	32·2	49·2	6·5	51·1	12·3

44	Loire-Atlantique	Pays-de-Loire	87	131	344	211·1	1437·1	24·2	48·5	6·7	89·6	10·2
45	Loiret	Centre-Val-de-Loire	51	60	244	100·8	682·9	26·4	48·6	7·9	82·6	13·2
46	Lot	Occitanie	8	4	273	33·2	173·2	38·9	48·4	8·1	40·8	14·7
47	Lot-et-Garonne	Nouvelle-Aquitaine	11	18	252	61·6	330·3	33·9	48·0	8·3	64·3	17·2
48	Lozère	Occitanie	3	0	237	14·8	76·3	33·5	49·6	5·1	0·1	14·3
49	Maine-et-Loire	Pays-de-Loire	66	98	325	113·8	815·9	27·3	48·7	7·5	76·1	11·7
50	Manche	Normandie	28	26	255	82·6	490·7	33·0	48·7	6	50·3	12·5
51	Marne	Grand-Est	60	39	342	69·1	563·8	26·1	48·4	7·5	77·6	14·4
52	Haute-Marne	Grand-Est	8	6	259	27·3	169·3	33·1	48·9	6·9	49·7	15·6
53	Mayenne	Pays-de-Loire	8	8	200	59·0	305·4	29·5	49·2	5·4	54·9	11·7
54	Meurthe-et-Moselle	Grand-Est	95	86	403	139·2	730·4	26·2	48·7	7·8	92·3	14·5
55	Meuse	Grand-Est	8	10	213	29·2	181·6	30·8	49·5	7·5	53·2	15·5
56	Morbihan	Bretagne	22	32	305	110·7	755·6	32·5	48·4	7	67·2	11·3
57	Moselle	Grand-Est	88	56	273	166·6	1035·9	27·4	48·9	7·9	88·7	14·6
58	Nièvre	Bourgogne-Franche Comté	12	16	233	29·3	199·6	38·3	48·0	7	46	15·8
59	Nord	Hauts-de-France	239	349	358	450·8	2589·0	23·2	48·3	10·5	95·4	19·2

60	Oise	Hauts-de-France	36	46	217	140·8	825·1	23·7	48·8	8·1	91·5	13·1
61	Orne	Normandie	16	12	242	45·4	276·9	34·1	48·8	7·5	33·8	16
62	Pas-de-Calais	Hauts-de-France	85	137	255	217·8	1452·8	25·8	48·5	9·9	93·6	19·8
63	Puy-de-Dôme	Auvergne-Rhône-Alpes	81	52	363	82·8	660·2	28·8	48·4	7	81·6	12·9
64	Pyrénées-Atlantiques	Nouvelle-Aquitaine	37	55	386	89·4	683·2	31·2	48·0	6·8	77·3	12·1
65	Hautes-Pyrénées	Occitanie	12	22	352	50·8	226·8	35·4	48·2	9	52	15
66	Pyrénées-Orientales	Occitanie	32	32	342	116·4	479·0	33·7	47·4	13·3	83·7	20·7
67	Bas-Rhin	Grand-Est	134	104	405	238·2	1132·6	25·5	48·5	6·8	87·2	13·1
68	Haut-Rhin	Grand-Est	70	68	303	216·5	763·2	27·0	49·0	7·8	88	13·1
69	Rhône	Auvergne-Rhône-Alpes	222	211	448	577·4	1876·1	22·3	48·1	7·2	97·7	13·9
70	Haute-Saône	Bourgogne-Franche Comté	12	12	236	43·5	233·2	31·1	49·1	7·4	52·9	14
71	Saône-et-Loire	Bourgogne-Franche Comté	26	22	262	63·9	547·8	33·7	48·5	7·4	62·5	13
72	Sarthe	Pays-de-Loire	21	27	235	90·3	560·2	29·4	48·5	8·1	80·5	13·4
73	Savoie	Auvergne-Rhône-Alpes	18	18	367	71·8	432·5	27·9	48·8	5·9	73	10·1
74	Haute-Savoie	Auvergne-Rhône-Alpes	34	41	303	188·8	828·4	22·7	49·1	6	92	9·2
75	Paris	Île-de-France	471	541	858	20459·7	2148·3	22·6	47·0	6·2	100	15·8

76	Seine-Maritime	Normandie	103	150	328	198·1	1243·8	26·9	48·0	9·2	86·8	14·7
77	Seine-et-Marne	Île-de-France	77	50	214	240·7	1423·6	20·5	48·5	6·7	98·7	11·6
78	Yvelines	Île-de-France	84	102	287	634·2	1448·6	22·6	48·5	6·2	100	9·7
79	Deux-Sèvres	Nouvelle-Aquitaine	8	18	236	62·1	372·6	30·9	48·8	5·8	43·2	12·4
80	Somme	Hauts-de-France	66	82	356	92·3	569·8	26·7	48·5	9·5	64·9	17·1
81	Tarn	Occitanie	24	35	299	67·4	387·9	32·5	48·0	8·5	56·9	15·5
82	Tarn-et-Garonne	Occitanie	18	20	264	70·6	262·6	28·8	48·9	9·4	77·2	17·2
83	Var	Provence-Alpes-Côte d'azur	60	42	337	179·8	1073·8	33·3	47·9	8·9	87·9	15·5
84	Vaucluse	Provence-Alpes-Côte d'azur	16	30	333	157·3	561·0	29·0	48·0	10·6	79·9	19·7
85	Vendée	Pays-de-Loire	18	22	219	101·7	683·2	32·1	48·6	6·3	49·7	9·6
86	Vienne	Nouvelle-Aquitaine	56	62	353	62·6	437·4	29·2	48·1	6·5	79·4	14·1
87	Haute-Vienne	Nouvelle-Aquitaine	26	72	413	67·2	370·8	32·3	47·5	7·4	77·1	15·3
88	Vosges	Grand-Est	8	12	254	61·2	359·5	32·7	48·6	8·7	55·9	15·7
89	Yonne	Bourgogne-Franche Comté	18	16	222	44·7	332·1	32·2	48·8	7·6	59·5	14·7
90	Territoire de Belfort	Bourgogne-Franche Comté	25	20	327	230·1	140·1	27·2	49·5	8·8	89·7	14·6
91	Essonne	Île-de-France	97	82	250	731·4	1319·4	20·5	48·9	6·5	100	12·9

92	Hauts-de-Seine	Île-de-France	210	138	399	9169·1	1613·8	20·7	47·5	6·3	100	12·2
93	Seine-Saint-Denis	Île-de-France	100	109	258	7076·9	1670·1	16·7	49·3	10·4	100	28·6
94	Val-de-Marne	Île-de-France	176	169	385	5738·9	1406·0	20·5	48·1	7·2	100	16·7
95	Val-d'Oise	Île-de-France	60	65	254	1001·9	1248·4	19·3	48·3	8·2	100	16·8
