# Supplementary material for: COVID-19 mortality with regard to healthcare services availability, health risks, and socio-spatial factors at department level in France: A spatial cross-sectional analysis
Source: PLoS One. 2021 Sep 17;16(9):e0256857. doi: 10.1371/journal.pone.0256857 (PMC8448369; doi:10.1371/journal.pone.0256857)
Supplement: S2 Table — (PDF) [file pone.0256857.s002.pdf]

S2 Table: Difference of the COVID-19 mortality rate in hospital between the first and second waves

Department number	Departments	COVID-19 mortality rate in hospital		Absolute difference	Relative difference (%)
		Wave 1 (Up to August 1st, 2020)	Wave 2 (From August 2nd to November 30th, 2020)		
**01**	Ain	15.98	29.38	13.40	83.81
**02**	Aisne	53.04	21.67	- 31.37	- 59.14
**03**	Allier	12.37	52.82	40.44	326.83
**04**	Alpes-de-Haute-Provence	7.87	30.27	22.40	284.62
**05**	Hautes-Alpes	12.70	57.85	45.15	355.56
**06**	Alpes-Maritimes	17.60	21.12	3.52	20.00
**07**	Ardèche	33.65	43.75	10.10	30.00
**08**	Ardennes	22.60	30.13	7.53	33.33
**09**	Ariège	1.31	11.81	10.50	800.00
**10**	Aube	43.88	25.81	- 18.07	- 41.18
**11**	Aude	15.83	13.95	- 1.88	- 11.86
**12**	Aveyron	8.62	29.10	20.48	237.50
**13**	Bouches-du-Rhône	27.87	44.43	16.56	59.44
**14**	Calvados	12.29	17.50	5.21	42.35
**15**	Cantal	6.30	16.11	9.80	155.56
**16**	Charente	3.73	5.74	2.01	53.85
**17**	Charente-Maritime	7.88	7.57	- 0.31	- 3.92
**18**	Cher	28.68	41.50	12.82	44.71
**19**	Corrèze	15.40	18.31	2.91	18.92
**2A**	Corse-du-Sud	46.91	28.34	- 18.58	- 39.60
**2B**	Haute-Corse	6.54	7.55	1.01	15.38
**21**	Côte-d'Or	12.04	26.66	14.62	121.43
**22**	Côtes-d'Armor	3.43	5.63	2.20	64.29
**23**	Creuse	27.99	14.46	- 13.53	- 48.34
**24**	Dordogne	24.78	39.38	14.60	58.91
**25**	Doubs	13.98	18.81	4.83	34.52
**26**	Drôme	33.07	16.77	- 16.30	- 49.30
**27**	Eure	4.85	4.30	- 0.55	- 11.36
**28**	Eure-et-Loir	29.55	16.01	- 13.55	- 45.83
**29**	Finistère	6.04	13.72	7.68	127.27
**30**	Gard	12.96	22.85	9.89	76.29
**31**	Haute-Garonne	5.21	15.06	9.85	189.04
**32**	Gers	12.10	14.21	2.10	17.39
**33**	Gironde	9.67	12.49	2.82	29.11
**34**	Hérault	10.46	23.98	13.52	129.27
**35**	Ille-et-Vilaine	8.13	11.92	3.79	46.59
**36**	Indre	36.38	16.12	- 20.26	- 55.70
**37**	Indre-et-Loire	14.37	13.71	- 0.66	- 4.60
**38**	Isère	12.10	43.80	31.70	262.09

**39**	Jura	23.27	37.23	13.96	60.00
**40**	Landes	3.16	10.92	7.77	246.15
**41**	Loir-et-Cher	19.22	17.08	- 2.14	- 11.11
**42**	Loire	32.43	58.97	26.55	81.85
**43**	Haute-Loire	7.93	35.70	27.77	350.00
**44**	Loire-Atlantique	11.41	12.73	1.32	11.59
**45**	Loiret	15.08	13.47	- 1.61	- 10.68
**46**	Lot	12.70	9.24	- 3.46	- 27.27
**47**	Lot-et-Garonne	3.33	10.60	7.27	218.18
**48**	Lozère	1.31	44.57	43.26	3,300.00
**49**	Maine-et-Loire	17.28	19.37	2.08	12.06
**50**	Manche	9.58	13.45	3.87	40.43
**51**	Marne	47.36	18.27	- 29.09	- 61.42
**52**	Haute-Marne	53.18	34.27	- 18.91	- 35.56
**53**	Mayenne	14.74	18.99	4.26	28.89
**54**	Meurthe-et-Moselle	49.29	19.99	- 29.30	- 59.44
**55**	Meuse	56.71	20.92	- 35.78	- 63.11
**56**	Morbihan	12.71	10.19	- 2.51	- 19.79
**57**	Moselle	81.48	25.97	- 55.51	- 68.13
**58**	Nièvre	14.03	22.04	8.02	57.14
**59**	Nord	25.72	34.26	8.54	33.18
**60**	Oise	50.42	16.24	- 34.18	- 67.79
**61**	Orne	14.45	24.92	10.47	72.50
**62**	Pas-de-Calais	21.96	25.67	3.72	16.93
**63**	Puy-de-Dôme	6.51	32.11	25.60	393.02
**64**	Pyrénées-Atlantiques	3.95	22.98	19.03	481.48
**65**	Hautes-Pyrénées	11.90	27.77	15.87	133.33
**66**	Pyrénées-Orientales	7.10	16.08	8.98	126.47
**67**	Bas-Rhin	59.16	18.28	- 40.88	- 69.10
**68**	Haut-Rhin	107.97	12.32	- 95.65	- 88.59
**69**	Rhône	35.39	44.03	8.64	24.40
**70**	Haute-Sâone	33.02	19.73	- 13.29	- 40.26
**71**	Sâone-et-Loire	36.69	56.04	19.35	52.74
**72**	Sarthe	14.99	13.57	- 1.43	- 9.52
**73**	Savoie	16.88	67.74	50.86	301.37
**74**	Haute-Savoie	20.16	40.20	20.04	99.40
**75**	Paris	82.67	32.96	- 49.71	- 60.14
**76**	Seine-Maritime	14.79	27.98	13.19	89.13
**77**	Seine-et-Marne	49.31	23.11	- 26.20	- 53.13
**78**	Yvelines	36.45	25.13	- 11.32	- 31.06
**79**	Deux-Sèvres	5.90	14.22	8.32	140.91
**80**	Somme	40.89	17.90	- 22.99	- 56.22
**81**	Tarn	5.93	20.62	14.69	247.83
**82**	Tarn-et-Garonne	2.28	26.27	23.99	1,050.00
**83**	Var	13.04	24.21	11.17	85.71
**84**	Vaucluse	6.77	52.05	45.28	668.42

**85**	Vendée	6.15	6.59	0.44	7.14
**86**	Vienne	9.14	16.23	7.09	77.50
**87**	Haute-Vienne	6.74	24.27	17.53	260.00
**88**	Vosges	73.99	28.37	- 45.62	- 61.65
**89**	Yonne	28.31	21.38	- 6.93	- 24.47
**90**	Territoire de Belfort	142.71	52.09	- 90.62	- 63.50
**91**	Essonne	40.78	27.89	- 12.88	- 31.60
**92**	Hauts-de-Seine	67.73	22.49	- 45.24	- 66.79
**93**	Seine-Saint-Denis	60.59	22.03	- 38.56	- 63.64
**94**	Val-de-Marne	85.99	34.07	- 51.92	- 60.38
**95**	Val-d'Oise	57.04	27.32	- 29.72	- 52.11
