# Supplementary material for: COVID-19 mortality with regard to healthcare services availability, health risks, and socio-spatial factors at department level in France: A spatial cross-sectional analysis
Source: PLoS One. 2021 Sep 17;16(9):e0256857. doi: 10.1371/journal.pone.0256857 (PMC8448369; doi:10.1371/journal.pone.0256857)
Supplement: S3 Table — (PDF) [file pone.0256857.s003.pdf]

S3 Table: Estimates effects of health services availability, socio-spatial factors, and health risk factors for COVID-19 pandemic mortality rate using the negative binomial regression model with Over-dispersion Correction

Wave 1 (up to 1 August 2020)

	Model 1	Model 2	Model 3	Model 4
Number of resuscitation beds (per 100,000 people)	**1.0069**** **(1.0029;1.0109)**	**1.0043** **(0.9994;1.0092)**	**1.0031** **(0.9976;1.0085)**	**1.0001** **(0.9960;1.0042)**
Physicians density (per 100,000 people)	**0.9965*** **(0.9935;0.9996)**	**0.9987** **(0.9957;1.0018)**	**0.9986** **(0.9955;1.0016)**	**1.0017** **(0.9993;1.0042)**
% People aged 60+		**0.9460*** **(0.9015;0.9927)**	**1.0169** **(0.9362;1.1046)**	**1.0811*** **(1.0115;1.1557)**
% Males		**1.6390*** **(1.0880;2.4692)**	**2.0123**** **(1.2714;3.1852)**	**1.6773**** **(1.1625;2.4201)**
% Urban population			**1.0144** **(0.9966;1.0324)**	**1.0078** **(0.9945;1.0213)**
Population density (log)			**1.1860** **(0.8991;1.5661)**	**1.3569**** **(1.0870;1.6940)**
Rate of poverty (per cent)			**1.0339** **(0.9683;1.1037)**	**0.9207**** **(0.8650;0.9800)**
Stand_Diabetes				**1.0947***** **(1.0687;1.1213)**
Stand_Chronic heart failure				**0.9850** **(0.8422;1.1519)**
Stand_Chronic respiratory diseases				**0.9702**** **(0.9489;0.9920)**
