# Supplementary material for: COVID-19 mortality with regard to healthcare services availability, health risks, and socio-spatial factors at department level in France: A spatial cross-sectional analysis
Source: PLoS One. 2021 Sep 17;16(9):e0256857. doi: 10.1371/journal.pone.0256857 (PMC8448369; doi:10.1371/journal.pone.0256857)
Supplement: S4 Table — (PDF) [file pone.0256857.s004.pdf]

S4 Table: Estimates effects of health services availability, socio-spatial factors, and health risk factors for COVID-19 pandemic mortality rate using the negative binomial regression model with Over-dispersion Correction

Wave 2 (between 1 August and 30 November 2020)

	**Model 1**	**Model 2**	**Model 3**	**Model 4**
Number of resuscitation beds (per 100,000 people)	**0.9996 (0.9976;1.0016)**	**0.9994 (0.9968;1.0020)**	**0.9997 (0.9968;1.0026)**	**0.9989 (0.9962;1.0017)**
Physicians density (per 100,000 people)	**1.0013 (0.9996;1.0030)**	**1.0020* (1.0002;1.0039)**	**1.0022* (1.0003;1.0040)**	**1.0028** (1.0010;1.0047)**
% People aged 60+		**0.9907 (0.9618;1.0205)**	**0.9966 (0.9421;1.0541)**	**0.9988 (0.9505;1.0496)**
% Males		**1.2803 (0.9859;1.6626)**	**1.3899 (0.9898;1.9517)**	**1.2595 (0.9368;1.6933)**
% Urban population			**1.0048 (0.9945;1.0153)**	**1.0035 (0.9942;1.0128)**
Population density			**0.9374 (0.7764;1.1320)**	**0.9474 (0.7955;1.1285)**
Rate of poverty (per cent)			**1.0224 (0.9849;1.0613)**	**1.0213 (0.9776;1.0669)**
Stand_Diabetes				**1.0148 (0.9962;1.0337)**
Stand_Chronic heart failure				**1.1452* (1.0207;1.2849)**
Stand_Chronic respiratory diseases				**0.9646*** (0.9508;0.9809)**
