# Supplementary material for: COVID-19 mortality with regard to healthcare services availability, health risks, and socio-spatial factors at department level in France: A spatial cross-sectional analysis
Source: PLoS One. 2021 Sep 17;16(9):e0256857. doi: 10.1371/journal.pone.0256857 (PMC8448369; doi:10.1371/journal.pone.0256857)
Supplement: S5 Table — (PDF) [file pone.0256857.s005.pdf]

S5 Table: Summary of parameter estimates of GWNBR models and assessing for spatial heterogeneity

All the period up to 30 November 2020 (Wave 1 + Wave 2)

Parameters	Minimum	1st Quartile	Median	3rd Quartile	Maximum	Interquartile (IQR)	Standard Error	Status
Intercept	-20.86843	-20.51730	-20.32608	-20.04047	-19.68764	0.47682	6.9904	Stationary
Number of resuscitation beds (per 100,000 people)	-0.00130	-0.00123	-0.00120	-0.00117	-0.00112	0.00006	0.0013	Stationary
Physicians density	0.00291	0.00295	0.00297	0.00299	0.00303	0.00004	0.0008	Stationary
% People aged 60+	0.02398	0.02568	0.02666	0.02738	0.02905	0.00170	0.0224	Stationary
% Males	0.40637	0.41374	0.41967	0.42358	0.43083	0.00984	0.1292	Stationary
% Urban population	0.00256	0.00281	0.00291	0.00303	0.00331	0.00022	0.0043	Stationary
Population density	0.14274	0.14295	0.14324	0.14358	0.14445	0.00062	0.0781	Stationary
Rate of poverty (per cent)	-0.02672	-0.02603	-0.02546	-0.02485	-0.02444	0.00118	0.0207	Stationary
Stand_Diabetes	0.05006	0.05079	0.05133	0.05180	0.05223	0.00101	0.0083	Stationary
Stand_Chronic heart failure	0.05688	0.06238	0.06847	0.07251	0.08119	0.01013	0.0536	Stationary
Stand_Chronic respiratory diseases	-0.03434	-0.03398	-0.03377	-0.03354	-0.03312	0.00044	0.0073	Stationary

Pseudo R^2 (pctdev)=0.6003, Adjusted R^2 =0.5444, BIC=867.4208, AIC=834.9871, AICC=839.1793, p=0.04348, t-critical = 2.05
