# Supplementary material for: COVID-19 mortality with regard to healthcare services availability, health risks, and socio-spatial factors at department level in France: A spatial cross-sectional analysis
Source: PLoS One. 2021 Sep 17;16(9):e0256857. doi: 10.1371/journal.pone.0256857 (PMC8448369; doi:10.1371/journal.pone.0256857)

Spatial disparity of the COVID-19 mortality rate in hospital according the number of intensive care beds per 1000.000 people at the department level in the first wave

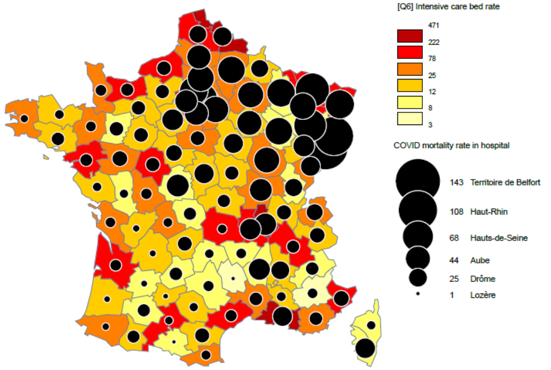

Spatial disparity of the COVID-19 mortality rate in hospital according the number of intensive care beds per 1000.000 people at the department level in the second wave

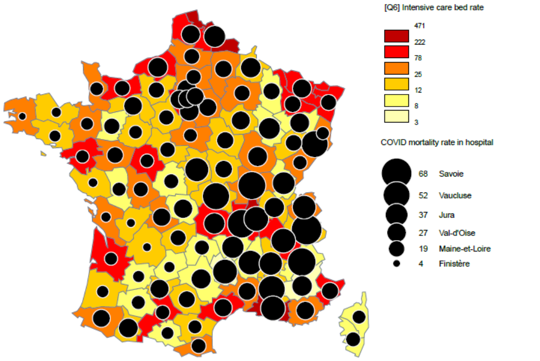

Spatial disparity of the COVID-19 mortality rate in hospital according the number of resuscitation beds per 1000.000 people at the department level in the first wave

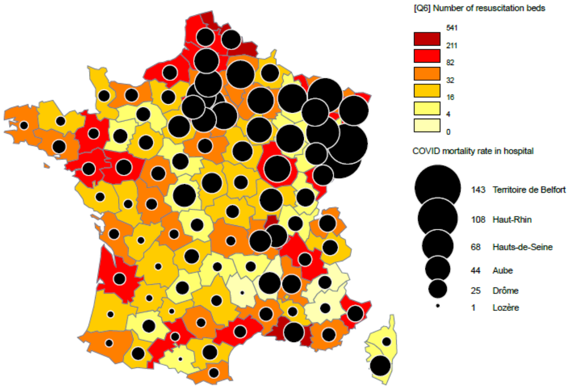

Spatial disparity of the COVID-19 mortality rate in hospital according the number of resuscitation beds per 1000.000 people at the department level in the second wave

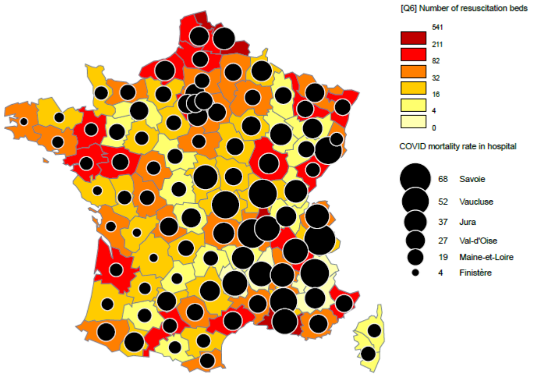

Spatial disparity of the COVID-19 mortality rate in hospital according the diabetes prevalence at the department level in the first wave

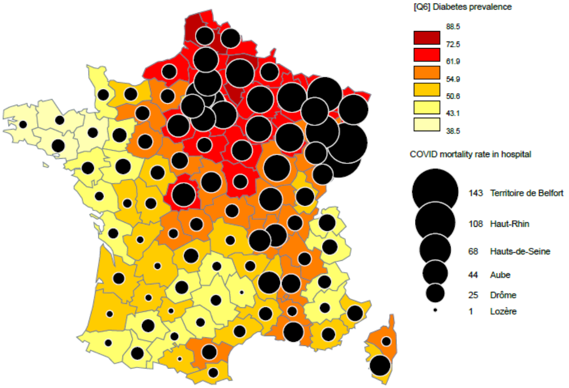

Spatial disparity of the COVID-19 mortality rate in hospital according the diabetes prevalence at the department level in the second wave

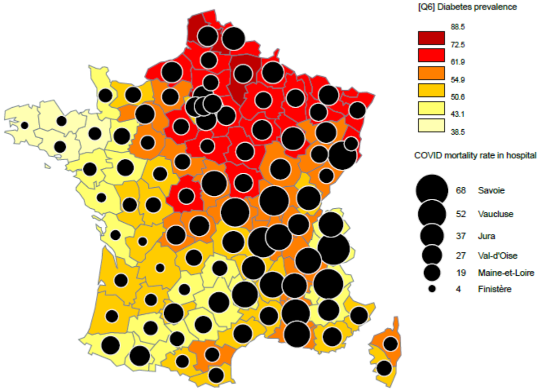

Supplement: S1 Fig — (PDF) [file pone.0256857.s006.pdf]
