# Supplementary material for: COVID-19 mortality with regard to healthcare services availability, health risks, and socio-spatial factors at department level in France: A spatial cross-sectional analysis
Source: PLoS One. 2021 Sep 17;16(9):e0256857. doi: 10.1371/journal.pone.0256857 (PMC8448369; doi:10.1371/journal.pone.0256857)

Spatial disparity of the COVID-19 mortality rate in hospital according the standardized rate of cardiovascular hospitalization at the department level in the first wave

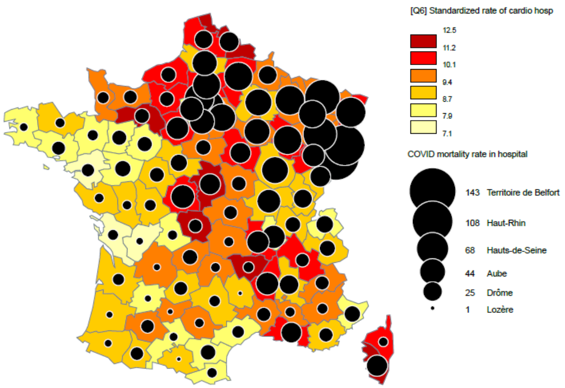

Spatial disparity of the COVID-19 mortality rate in hospital according the standardized rate of cardiovascular hospitalization at the department level in the second wave

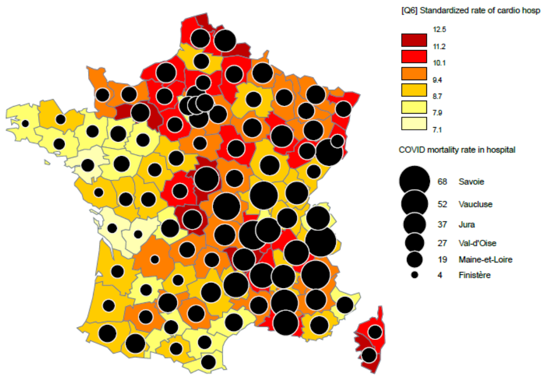

Spatial disparity of the COVID-19 mortality rate in hospital according the urbanization rate at the department level in the first wave

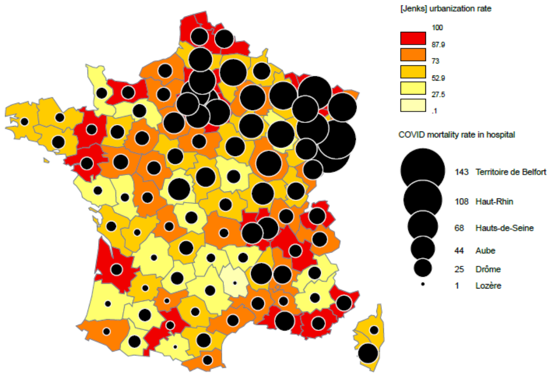

Spatial disparity of the COVID-19 mortality rate in hospital according the urbanization rate at the department level in the second wave

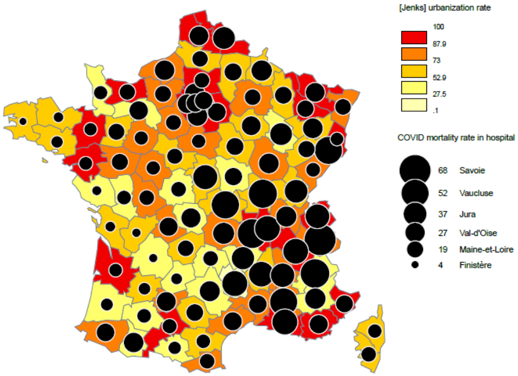

Spatial disparity of the COVID-19 mortality rate in hospital according the population density at the department level in the first wave

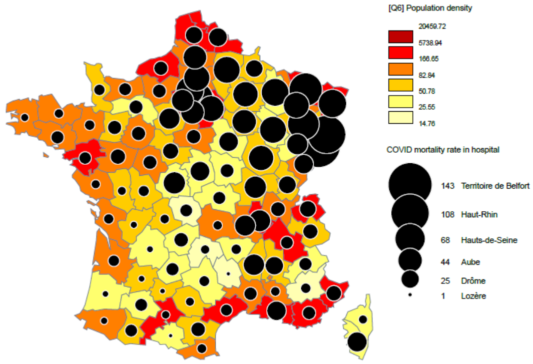

Spatial disparity of the COVID-19 mortality rate in hospital according the population density at the department level in the second wave

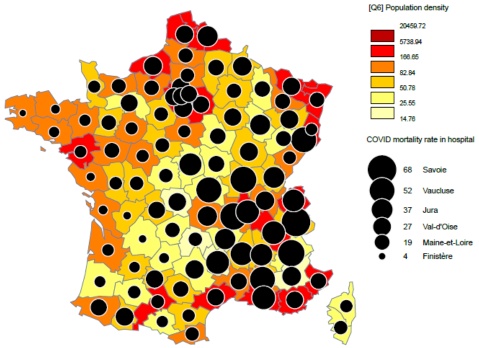

Supplement: S2 Fig — (PDF) [file pone.0256857.s007.pdf]
