# Supplementary figures and images for: COVID-19 mortality with regard to healthcare services availability, health risks, and socio-spatial factors at department level in France: A spatial cross-sectional analysis
Source: PLoS One. 2021 Sep 17;16(9):e0256857. doi: 10.1371/journal.pone.0256857 (PMC8448369; doi:10.1371/journal.pone.0256857)

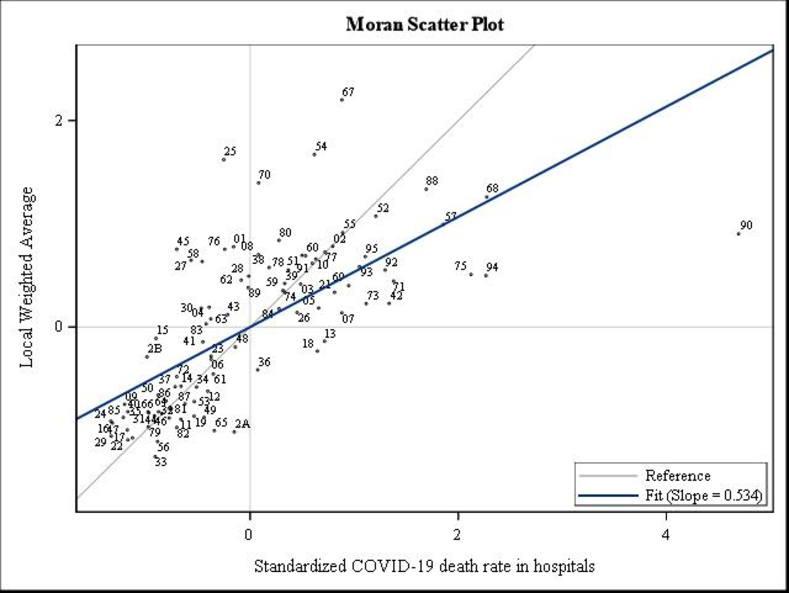

Supplement: S3 Fig — (TIF) [file pone.0256857.s008.tif]
